# Association between medication literacy and medication adherence and the mediating effect of self-efficacy in older people with multimorbidity

**DOI:** 10.1186/s12877-023-04072-0

**Published:** 2023-06-19

**Authors:** Wenna Wang, Wenyan Luan, Zhenxiang Zhang, Yongxia Mei

**Affiliations:** 1 Academy of Medical Sciences, Zhengzhou University, Zhengzhou, Henan China; 2 School of Nursing and Health, Zhengzhou University, No. 100, Kexue Road, Zhongyuan District, Zhengzhou, Henan China

**Keywords:** Elderly, Multimorbidity, Medication literacy, Medication adherence, Self-efficacy

## Abstract

**Background:**

Multimorbidity has a significant impact on public health and primary care. Medication adherence is recognized as the most effective measure for managing and preventing multimorbidity. Studies have shown that medication literacy has a positive effect on medication adherence in patients with multimorbidity. However, limited knowledge exists regarding the underlying mechanisms of this relationship in older adults with multimorbidity. Therefore, the aim of this study was to investigate the mediating role of self-efficacy in the association between medication literacy and medication adherence in this population.

**Methods:**

This study employed a cross-sectional design and convenience sampling method to survey older patients with multimorbidity in six communities in Zhengzhou, China, from July 12, 2021, to December 15, 2021. Participants were assessed using a demographic questionnaire, the Chinese Version of the Medication Literacy Scale (C-MLS), the Self-Efficacy for Appropriate Medication Use Scale (SEAMS), and the Chinese Version of the Morisky Medication Adherence Scale-8 (C-MMAS-8). Data were analyzed using descriptive statistics, t-tests, one-way analysis of variance, Pearson correlation analysis, and mediation analysis.

**Results:**

A total of 350 elderly patients met the inclusion criteria, and 328 valid questionnaires were collected. The mean age of the participants was 74.90 ± 7.37 years, with a slightly higher proportion of males (55.8%) than females (44.2%). The mean score for medication adherence was 4.85 ± 1.57, indicating poor medication adherence among the participants. Medication adherence scores varied significantly among participants of different ages, education levels, employment statuses and kinds of medication (p < 0.01). Scores for medication literacy and self-efficacy showed a significant positive correlation with medication adherence scores (all p < 0.001). The standardized coefficient for the total effect and direct effect of medication literacy on medication adherence was 0.268 (95% CI: 0.201, 0.335) and 0.187 (95% CI: 0.123, 0.252), respectively. After introducing self-efficacy into the model, the standardized coefficient for the indirect effect was 0.081 (95% CI: 0.049, 0.120), indicating that self-efficacy partially mediated the relationship between medication literacy and medication adherence, accounting for 30.22% of the total effect.

**Conclusion:**

This study might suggest that medication literacy indirectly affected medication adherence in older people with multimorbidity through self-efficacy. Health care providers should be aware of the importance of improving medication literacy and implement strategies aimed at increasing self-efficacy to achieve the goal of improving medication adherence in older adults with multimorbidity.

## Background

Multimorbidity, defined as having two or more chronic diseases, has a major impact on public health and primary care [1, 2]. With changing lifestyles and the increasing aging process, multimorbidity has become a common phenomenon among the elderly population. Recent systematic studies have shown that the prevalence of multimorbidity ranges from 23 to 33%, and it further increases with age [3]. A systematic review indicated that the prevalence of multimorbidity in older individuals ranged from 55 to 98% [4]. As the prevalence of multimorbidity in older people continues to rise, reflecting unhealthy lifestyles and global population ageing, it poses a significant challenge to healthcare systems. As reported from the existing study, patients with multimorbidity were associated with increased healthcare costs, poorer clinical outcomes, and increased disability and mortality [3, 5]. Therefore, it is crucial to allocate more research and resources to address the needs of the elderly population with multimorbidity.

Medication adherence, defined as patients taking their medications as prescribed, is essential to achieve optimal disease control [6]. Recent guidelines and studies have highlighted the importance of medication adherence as a cornerstone for managing and preventing long-term complications in patients with multimorbidity [7, 8]. Timely, regular, and long-term adherence to medication could effectively improve symptoms and further control the development of the disease. Some studies [7, 9, 10] have also recognized that higher medication adherence could effectively improve multimorbidity patient’s quality of life, decrease complications and mortality. However, medication adherence is especially difficult to ensure, especially in the elderly. Medication nonadherence is highly prevalent among older adults, more than 55% of whom have multimorbidity [4]. A recent longitudinal medication adherence survey estimated that 65% of older adults with multimorbidity did not take their prescribed medications [11]. Previous studies have found that older adults are particularly vulnerable to nonadherence due to polypharmacy associated with multimorbidity [12].

Correct and adequate medication literacy and a positive attitude toward disease treatment are the basis of sustained medication adherence. Medication literacy refers to the ability of individuals to acquire, understand, communicate, calculate and process medication information so as to make informed medication and health decisions, and then to take medication safely and effectively [13]. Several studies have demonstrated that participants with higher levels of medication literacy tend to exhibit greater medication adherence in patients with chronic conditions [14, 15]. Therefore, it is important to assess the current level of medication literacy and its impact on medication adherence among older patients with multimorbidity. However, the specific mechanism mediating the relationship between medication literacy in older patients with multimorbidity and their adherence to medication regimens is unclear and requires further research.

Fisher proposed the information-motivation-behavioral skills model (IMB) [16], which suggests that behaviors are influenced by information, motivation, and behavioral skills. In this model, self-efficacy plays a crucial role as an important factor in behavioral skills. Specifically, health literacy can directly impact an individual’s medication adherence behavior or indirectly influence it by enhancing self-efficacy. Self-efficacy, defined as the confidence in one’s ability to perform tasks such as taking medications, is a significant determinant of medication adherence [17, 18]. Numerous studies have confirmed the direct effects of self-efficacy on medication adherence in patients with chronic diseases or multimorbidity [19–21]. Moreover, the self-efficacy of patients with chronic diseases can be influenced by their mastery of disease-related knowledge, such as medication literacy [22, 23]. For instance, a recent study in hypertensive patients demonstrated a significant positive association between medication literacy and self-efficacy (r = 0.408, p < 0.001), and further revealed that self-efficacy partially mediated the relationship between medication literacy and adherence to antihypertensive regimens [24]. Therefore, self-efficacy may serve as an important mediator between medication literacy and medication adherence in patients with multimorbidity. By understanding the specific impact and mechanisms of medication literacy on medication adherence, it becomes possible to develop more targeted interventions to promote medication adherence and improve health outcomes among older patients with multimorbidity. Thus, the objective of this study was to examine the relationship between medication literacy, self-efficacy, and medication adherence, as well as explore the mediating role of self-efficacy between medication literacy and medication adherence in older patients with multimorbidity.

## Methods

### Study design and setting

This was a cross-sectional study reported in accordance with the Strengthening the Reporting of Observational Studies in Epidemiology (STROBE) recommendations [25]. This study was conducted in six communities in Zhengzhou, China, from July 12, 2021, to December 15, 2021.

### Participants and procedure

The participants of this study were community-dwelling older patients with multimorbidity. Multimorbidity is defined as having two or more chronic conditions [4]. As suggested from the relevant literature on the multimorbidity among the elderly in China, The most common six types of chronic diseases include hypertension, coronary heart disease, chronic pulmonary disease, hyperlipidemia, diabetes mellitus, and stroke. The prevalence rates for these diseases are 21.3%, 12.66%, 10.9%, 9.67%, 6.10%, and 2.71%, respectively [12]. The inclusion criteria for participants were as follows: (1) age 60 years or older; (2) presence of at least two out of the six chronic diseases mentioned above; (3) use of at least one prescribed medication for a chronic condition for a minimum of 3 months prior to study inclusion; (4) ability to speak and understand Chinese; (5) capable of providing informed consent to participate in the study. Patients with cognitive impairment (Mini-Mental State Examination score < 27) were excluded from the study as they may not be able to provide valid responses to the questionnaires.

The sample size was calculated using G-power version 3.1, assuming a power of 0.95. The effect size was set at a medium value of 0.30, and a two-sided test was conducted at a significance level of 0.05. Therefore, the estimated sample size for this study was no less than 134. With the assistance of community health workers, two trained researchers contacted participants in advance to schedule appointments, either via phone or walk-in visits. The survey location was chosen based on participants’ preferences and included their homes, community health service centers, and nearby parks. The study’s purpose, research process, and participants’ rights were thoroughly explained to potential participants upon initial contact. Upon providing informed consent, participants were asked to complete the questionnaires. For those who were illiterate, had blurred vision, or faced other challenges, the investigator read the questions aloud one by one and recorded the participant’s chosen response.

### Outcome variables

#### Participant characteristics

Participant characteristics were collected through questionnaires, which included age, gender, education, marital status, employment status, number of chronic diseases, duration of multimorbidity status and medication frequency.

#### Chinese Version of the Medication Literacy Scale (C-MLS)

This scale was originally developed by Sauceda et al. [26] from the University of Texas at El Paso in the United States and is available in both English and Spanish. The scale contains four simulation cases with 14 items having a dichotomy scoring system (1 = correct; 0 = incorrect). The scale has a full score of 14. According to the discrimination of calculation formula of the distinction degree, which was used in the educational statistics, the scores were divided into three groups: adequate literacy (> 10), moderate literacy (4 ~ 10), and inadequate literacy (< 4). Higher scores indicate better patient medication literacy. Zheng et al. [27] translated and revised the scale into the Chinese version of the Medication Literacy Scale. The Chinese version of the scale was a one-dimensional medication literacy assessment scale with a retest reliability of 0.885, a fractional half reliability of 0.840 and a Kuder-Richardson 20 (K-R 20) of 0.820.

#### Self-efficacy for appropriate medication use scale (SEAMS)

The scale was developed by Risser et al. [18] for patients with chronic diseases and could be used to measure self-efficacy of appropriate medication use in patients with chronic diseases. The scale contains 2 dimensions (self-efficacy for taking medications under difficult circumstances and taking medications under uncertain circumstances), with a total of 13 items, requiring participants to report their confidence in taking medication under 13 situations (1 = not confident, 2 = somewhat confident, and 3 = very confident). The total score of the scale is the sum of the scores of each item, ranging from 13 to 39 points. Higher scores indicate higher levels of self-efficacy for appropriate medication. The reliability and validity of the Chinese version Self-Efficacy for Appropriate Medication Use Scale were evaluated in stroke survivors [28] with a Cronbach’s α coefficient of 0.934, a test-retest reliability of 0.932, and a scale-level content validity index (S-CVI) of 0.913.

#### Chinese version of the morisky medication adherence scale-8 (C-MMAS-8)

The MMAS-8 was originally developed by Morisky et al. [29], which is a concise, pragmatic and cost-effective self-administered measure that is primarily used to assess medication adherence levels. The scale consists of 8 items and is confirmed to have good reliability and validity in patients with hypertension. The Cronbach’s alpha coefficient of this scale was 0.83. In this scale, yes and no are the answer options for seven items, and the last question is answered on a 5-point Likert scale. The total score on this scale ranges from 0∼8. Higher scores represent better adherence to hypertensive medication. Morisky’s suggested cut-off point of 6 was applied: MMAS score < 6 (low adherence), score = 8 (high adherence), and score ≥ 6 and < 8 (medium adherence). The Chinese version of the MMAS-8 (C-MMAS-8) was translated by Yan [30]. Good reliability and validity (Cronbach’s α = 0.77, pretest-posttest correlation coefficient = 0.88) were identified in Chinese myocardial infarction patients. Every item of the Chinese version of MMAS-8 that was used in the present study was not different from the original English version, except for language differences.

### Statistical analysis

Data processing and analysis were conducted using the software package SPSS for Windows, version 21.0. Continuous variables with a normal distribution were described using means and standard deviations (mean ± SD), while categorical variables were presented as numbers or percentages. The scores of medication literacy, self-efficacy for appropriate medication use, and medication adherence among older multimorbidity patients with different demographic characteristics were compared using independent-sample t-tests or analysis of variance (ANOVA). Pearson correlation analysis was performed to determine the correlations among medication literacy, self-efficacy for appropriate medication use, and medication adherence. For testing the mediation model with the model-4 setup, the SPSS PROCESS procedure was utilized in this study. The bias-corrected bootstrapping procedure, with 5,000 repeated samplings, was employed to establish a 95% confidence interval (CI). If the CI did not include zero, the mediating effect was considered significant. Conversely, if the CI included zero, the mediating effect was considered insignificant [31].

## Results

A total of 350 elderly patients with multimorbidity participated in this study. However, 8 patients chose to drop out during the survey due to the perceived length of the questionnaire, and 14 patients completed questionnaires that were subsequently excluded due to missing key information, such as gender. As a result, 328 valid questionnaires were included, yielding a response rate of 93.71%. The characteristics of the participants are presented in Table 1. The mean age of the participants was 74.90 ± 7.37 years, with a slightly higher proportion of males (55.8%) than females (44.2%). Approximately 80% of the participants lived with their spouses, and the majority of them were either retired or unemployed (69.8%). Regarding medication adherence, 25 individuals (7.6%) demonstrated high adherence, 54 (16.5%) had moderate adherence, and 249 (75.9%) exhibited low adherence.

**Table 1:** Characteristics of participants in this study (n = 328)

Variable	n	%	Mean		SD	Range
Age, years			74.90	7.37		60–95
≤ 75	167	50.9				
>75	161	49.1				
Gender						
Male	183	55.8				
Female	145	44.2				
Education						
Primary school and below	102	31.1				
Junior high school	128	39.0				
High school or junior college	88	26.8				
Bachelor degree or above	10	3.0				
Marital status						
Have a spouse	262	79.9				
No spouse	66	20.1				
Employment status						
Retired or unemployed	229	69.8				
On-the-job	99	30.2				
Number of chronic diseases						
2	130	39.6				
3	112	34.2				
4~	86	26.2				
Duration of multimorbidity status, years						
0~	47	14.3				
5~	54	16.5				
10~	98	29.9				
15~	129	39.3				
Kinds of medication						
1~	138	42.1				
3~	159	48.5				
5~	31	9.5				
Medication frequency						
Once a day	18	5.5				
Twice a day	100	30.5				
Three or more times a day	210	64.0				

SD standard deviation

The scores of the three instruments in this study are shown in Table 2. The mean total score of C-MLS was (7.36 ± 2.55) with a range of 1 to 13 indicating moderate medication literacy. The scores on the SEAMS ranged from 13 to 36 points, with a mean score of (25.00 ± 4.58) points. The mean medication adherence score was (4.85 ± 1.57) with a range of 1 to 8, indicating poor medication adherence among the participants.

**Table 2:** Scores of medication literacy, self-efficacy, and medication adherence for participants (n = 328)

Self-report instrument	Mean	SD	Range
C-MLS	7.36	2.55	1–13
SEAMS	25.00	4.58	13–36
Under difficult circumstances	12.73	2.45	6–18
Uncertain circumstances	12.27	2.42	7–18
C-MMAS-8	4.85	1.57	1–8

SD standard deviation, C-MLS Chinese Version of the Medication Literacy Scale, SEAMS Self-Efficacy for Appropriate Medication Use Scale, C-MMAS-8 Chinese Version of the Morisky Medication Adherence Scale-8.

Participants with different ages, education levels, employment status, and kinds of medication had significantly different scores of medication adherence (p < 0.01). Correlation analysis between medication literacy, self-efficacy and medication adherence showed that both medication literacy scores and self-efficacy scores were positively correlated with medication adherence scores (r > 0.500, p < 0.01) (Table 3**and** Table 4).

**Table 3:** T/ANOVA tests of medication adherence and participant characteristics (n = 328)

Variable	Mean (SD)	F/t	p
Age, years			
≤ 75	68.87(4.27)	2.718^a^	0.007
>75	81.16(3.86)		
Gender		1.641^a^	0.102
Male	4.98(1.52)		
Female	4.70(1.62)		
Education		29.454^b^	< 0.001
Primary school and below	3.98(1.55)		
Junior high school	4.85(1.12)		
High school or junior college	5.89(1.53)		
Bachelor degree or above	4.85(1.81)		
Marital status		-1.452^a^	0.148
Have a spouse	4.91 (1.54)		
No spouse	4.63(1.70)		
Employment status		-4.662^a^	< 0.001
Retired or unemployed	5.11(1.53)		
On-the-job	4.26(1.50)		
Number of chronic diseases		1.679^b^	0.188
2	5.00(1.56)		
3	4.64(1.59)		
4~	4.92(1.56)		
Duration of multimorbidity status, years		0.856^b^	0.464
1~	5.18 (1.52)		
5~	4.88 (1.39)		
10~	4.77(1.60)		
15~	4.79 (1.64)		
Kinds of medication		10.941^b^	< 0.001
1~	5.32(1.64)		
3~	4.52(1.39)		
5~	4.51(1.70)		
Medication frequency		3.229^b^	0.410
Once a day	4.14(1.92)		
Twice a day	5.10(1.38)		
Three or more times a day	4.80(1.61)		

a = Independent Samples T-test, b = Chi-square test

**Table 4:** Correlations analysis between medication adherence and medication literacy and self-efficacy for appropriate medication use (n = 328)

Variable	Medication adherence	
	r	p
Medication literacy(C-MLS)	0.560	< 0.01
Self-efficacy(SEAMS)	0.564	< 0.01
Under difficult circumstances	0.522	< 0.01
Under uncertain circumstances	0.536	< 0.01

C-MLS Chinese Version of the Medication Literacy Scale, SEAMS Self-Efficacy for Appropriate Medication Use Scale.

PROCESS version 3.3 was used in this study to analyze the mediating effect between medication adherence and medication literacy (Table 5). Model 1 showed a significant explanatory power of medication literacy on medication adherence after controlling for demographic variables (β = 0.27, p < 0.001). Model 2 showed that medication literacy (β = 0.64, p < 0.001) had a significant explanatory power for self-efficacy. Model 3 considered the explanatory power of medication literacy and self-efficacy (β = 0.19, p < 0.001 and β = 0.13, p < 0.001) on medication adherence. In summary, the two variables have significant explanatory power on medication adherence. The standardized coefficient for the total effect and direct effect of medication literacy on medication adherence was 0.268 (95% CI: 0.201, 0.335) and 0.187 (95% CI: 0.123, 0.252), respectively. After introducing the mediating variable self-efficacy into the model, the standardized coefficient of the indirect effect is 0.081 (95% CI:0.049,0.120), which was statistically significant, indicating that self-efficacy had a partial mediating effect, accounting for 30.22% of the total effect.

**Table 5:** Mediation analysis of medication adherence and medication literacy for participants (n = 328)

Variable	Medication adherence						Self-efficacy		
	Model 1			Model 3			Model 2		
	β	SE	p	β	SE	p	β	SE	p
Medication literacy	0.27	0.03	< 0.001	0.19	0.03	< 0.001	0.64	0.11	< 0.001
Self-efficacy				0.13	0.02	< 0.001			
Age	-0.01	0.01	0.34	-0.01	0.01	0.31	-0.01	0.03	0.94
Education	0.26	0.10	0.01	0.19	0.10	0.04	0.59	0.32	0.07
Employment status	0.26	0.167	0.12	0.15	0.15	0.35	0.89	0.53	0.10
Kinds of medication	-0.29	0.11	0.01	-0.2533	0.10	0.02	-0.32	0.36	0.38
	**R**^**2**^ **= 0.35**	** F = 35.15**	**< 0.001**	**R**^**2**^ **= 0.46**	** F = 45.32**	**< 0.001**	**R**^**2**^ **= 0.22**	** F = 5.63**	**< 0.001**
	**Effect**	**SE**	**p**	**95% CI**		**%** ^**b**^			
Total effect	0.268	0.034	< 0.001	0.201	0.335	100			
Direct effect	0.187	0.033	< 0.001	0.123	0.252	69.98			
Indirect effect	0.081	0.018		0.049	0.120	30.22			

SE standard error, β Standardized coefficient, CI confidence interval.

^b^ Percentage of effect/total effect.

## Discussion

Taking medication as prescribed by a physician is the most effective measure for the treatment of chronic diseases [32]. Scores on the C-MMAS-8 in this study revealed poor medication adherence among the participants, with 75.9% of older adults with multimorbidity demonstrating low medication adherence. This percentage is higher compared to other studies conducted worldwide on populations with chronic diseases [33, 34]. Besides, age, education level, employment status, and kinds of medication were significantly associated with medication adherence, which echoes previous research [35]. This study showed that the older the elderly were, the lower their medication adherence. And this result may further confirm that older patients with multimorbidity are more likely to be non-adherent to their medications than younger patients [36]. And patients with higher education levels had better medication adherence. Additionally, retired or unemployed individuals demonstrated better medication adherence compared to those who were employed. Furthermore, the wider variety of medications taken by older adults contributed to lower adherence rates. These findings highlight the need for accurately targeted medication adherence interventions based on the specific characteristics of older patients. Given that medication adherence is a complex and multifaceted process, future studies should adopt a mixed-methods approach to further explore the comprehensive factors underlying medication non-adherence in elderly patients with multimorbidity. This will aid in the development of more targeted medication intervention programs for the elderly population.

The results demonstrated a significant positive correlation between medication literacy, self-efficacy, and medication adherence in older adults with multimorbidity. Similar findings have been reported in previous studies involving patient groups, such as hypertensive patients [37]. Specifically, patients with lower medication literacy exhibited lower medication adherence rates. This can be attributed to the fact that a lower level of medication literacy increases the likelihood of misunderstanding the disease, consequently leading to noncompliance with medication-related behaviors. Thus, it is crucial to assess medication literacy and develop effective interventions aimed at improving medication literacy in older adults with multimorbidity [38]. It is worth emphasizing that improving self-efficacy is an important intervention to improve medication adherence in clinical care. Healthcare workers can provide information on medication management and individualized guidance, encourage and assist in solving individual problems, and create successful experiences related to implementing proper medication. And older multimorbidity patients can also join peer support groups to share medication adherence tips and techniques with fellow patients to help them feel more confident about managing their medications. A systematic review has indicated that self-management interventions and electronic health interventions may be effective in improving medication adherence among older individuals with multimorbidity [35].

This study explored the mediating effect of self-efficacy on medication literacy and medication adherence in older people with multimorbidity. After controlling for patient characteristics, self-efficacy had a partial mediating effect on the relationship between medication literacy and medication adherence, accounting for 30.22% of the total effect. A possible explanation for this finding is that having optimal medication literacy is essential for multimorbidity patients to understand and administer different medications correctly and effectively. On the other hand, higher self-efficacy convinces patients that they have the ability to consistently adhere to their medication regimen throughout their lifetime. Conversely, when patients are kept in the dark about the medication they are taking, they have less confidence in the administration of the medication, resulting in poor medication adherence. The KABP Model also suggests that mastery of knowledge could stimulate a person’s self-efficacy, thus enabling the person to adopt healthy behaviors [39]. Therefore, self-efficacy is a vital mediating predictor of medication adherence. And for older multimorbidity patients with low medication literacy, self-efficacy should be focused on in order to promote their medication adherence. And some social cognitive and behavioral therapies in psychological treatment could be incorporated to improve self-efficacy for older multimorbidity patients to improve their medication adherence. In addition, the implementation of theoretical frameworks along with a patient-centred perspective is encouraged to provide by clinicians to enhance the patient’s medication adherence in future studies [40].

The limitations of this study should be acknowledged. First, a convenience sample of older participants with multimorbidity was employed which was drawn only from Zhengzhou, China. Whether and to what extent these participants can represent other older people with multimorbidity population in China requires a thorough investigation. Second, due to the complexity of multimorbidity population, only the six most common chronic diseases were selected to limit the inclusion criteria in this study, which may limit the generalizability of the findings. In addition, all variables in this cross-sectional study were collected in a questionnaire survey, thus it was unable to determine the continuous changes in medication literacy, self-efficacy and medication adherence. Continuous follow-up investigations should be carried out on older patients with multimorbidity in the future to clarify the influencing mechanism of the relationship between variables over time.

## Conclusions

This study provides initial evidence for older patients diagnosed with multimorbidity in mainland China, highlighting the partially significant mediating effect of self-efficacy on the relationship between medication literacy and medication adherence. Given the high prevalence of poor medication adherence among older patients with multimorbidity, enhancing medication literacy and self-efficacy may contribute to improving medication adherence in this population. Therefore, healthcare providers should recognize the significance of assessing and promoting medication literacy and implement strategies aimed at enhancing self-efficacy. These efforts can help achieve the goal of enhancing medication adherence among older patients with multimorbidity.

## Data Availability

The data used to support the findings of this study are available from the corresponding author upon request.

## Abbreviations

C-MLS: Chinese Version of the Medication Literacy Scale
SEAMS: Self-Efficacy for Appropriate Medication Use Scale
C-MMAS-8: Chinese Version of the Morisky Medication Adherence Scale-8
K-R 20: Kuder-Richardson 20
S-CVI: scale-level content validity index
SD: standard deviation
SE: standard error
β: Standardized coefficient
CI: confidence interval
